# Characterization and Adsorption Behavior of Strontium from Aqueous Solutions onto Chitosan-Fuller’s Earth Beads

**DOI:** 10.3390/healthcare7010052

**Published:** 2019-03-26

**Authors:** Shameem Hasan, A. Rafi M. Iasir, Tushar K. Ghosh, Bhaskar Sen Gupta, Mark A. Prelas

**Affiliations:** 1 Perma-Fix Environmental Services, Inc., 8302 Dunwoody Place, Suite 250, Atlanta, GA 30350, USA; shm_ha@yahoo.com; 2 Nuclear Science and Engineering Institute, University of Missouri, Columbia, MO 65211, USA; ai9tb@mail.missouri.edu (A.R.M.I.); ghosht@missouri.edu (T.K.G.); 3 Chemical Engineering, University of Missouri, Columbia, MO 65211, USA; 4 Water Academy, School of Energy, Geoscience Infrastructure and Society, Heriot-Watt University, Edinburgh EH14 4AS, UK; B.SenGupta@hw.ac.uk; 5 Electrical Engineering and Computer Science, University of Missouri, Columbia, MO 65211, USA

**Keywords:** chitosan, fuller’s earth, strontium, adsorption, ion-exchange, kinetics

## Abstract

Fuller’s earth spherical beads using chitosan as a binder were prepared for the removal of strontium ions from aqueous solution. The adsorbents were characterized by scanning electron microscopy (SEM) and transmission electron microscopy (TEM), which revealed the porous nature of the beads. The Brunauer–Emmett–Teller (BET) surface area of the beads was found to be 48.5 m^2^/g. The adsorption capacities of the beads were evaluated under both batch and dynamic conditions. The adsorption capacity was found to be ~29 mg/g of adsorbent at 298 K when the equilibrium concentration of strontium in the solution was 925 mg/L at pH 6.5. The X-ray photoelectron spectroscopy (XPS) data suggest that strontium uptake by the beads occurs mainly through an ion-exchange process. Kinetic data indicate that the sorption of strontium onto the beads follows anomalous diffusion. Thermodynamic data suggest that the ion-exchange of Sr^2+^ on the bead surface was feasible, spontaneous and endothermic in nature.

## 1. Introduction

Radioactive wastewater produced from the nuclear fuel cycle is a serious environmental concern due to its radiological and toxicological effect on the eco-system and human health. Strontium is one of the major radionuclides that are present in radioactive wastewater, which is, in general produced during the nuclear fission process. Strontium is a pure beta emitter of 0.546 MeV with a half-life of 28.8 years and is commonly found in many groundwater systems migrating from historic nuclear waste storage sites [1,2]. Once released in to the environment, strontium has the potential to percolate through underlying soil into groundwater and remain as a major contaminant for around ten half-lives [3,4,5]. Strontium preferentially adheres to soil particles and, over a long period of time, it can remain primarily in weakly bound surface complexes [3]. Strontium-90 chemically resembles calcium and its effective half-life is approximately 18 years [6]. Once ingested, it can substitute calcium in the bone structure of living organisms and acts as a long-term source of the irradiation of bone marrows that makes strontium-90 one of the most dangerous radionuclides to human health [7,8]. Therefore, the removal of strontium among all other contaminants from nuclear facility-related wastewaters is of great interest because of its relatively long half-life and high radiological toxicity. A number of methods including chemical precipitation, evaporation, solvent extraction, membrane and ion-exchange processes have been reported for the removal of radionuclides from low and medium strength effluents with a varying degree of success [9,10]. Among these methods, the ion-exchange method appears to be an effective and promising means of treating radioactive wastes. A variety of ion-exchange resins, for instance, SrTreat^®^ (Fortum Nuclear Services, Espoo, Finland) and crystalline silicotitanate have been investigated for the selective removal of strontium from nuclear waste solutions [11,12,13]. Although these sorbent materials performed better with high conductivity effluents, the cost of these sorbents could be a major factor for treatment of low-level wastewater [14]. The review paper by DiGisie et al., 2016 outlined the characteristic and adsorption capacities of low-cost adsorbents for wastewater treatment [15]. The affinity of sorbents in removing various pollutants, their applications on real wastewater, costs and considerations on their reuse after adsorption process has been discussed to a greater extent.

In general, the radionuclide Sr^2+^ adsorbs to a wide range of aluminosilicates, Fe oxides and other soil minerals via weakly bound surface complexes [3]. Several researchers also used natural sorption materials, such as bentonite, natural zeolite, gibbsite, mineral mixture, activated carbon, clinoptilolite, and attapulgite, as low-cost options for the removal of radionuclide from liquid wastes [16,17,18,19,20]. The adsorption capacity of various materials for strontium is summarized in Table 1.

In recent years, the natural clay mineral based low-cost adsorbents have become attractive again for water purification and radioactive waste management [16]. For example, natural clay materials such as attapulgite and bentonite have been used as back fill and buffer material in waste repositories to control the spreading of radionuclides [1,26]. The present work describes the preparation of low-cost adsorbents by dispersing natural materials such as Fuller’s earth onto the chitosan matrix. Fuller’s earth is considered a clay material, which mainly consists of hydrated aluminum silicate, containing a small proportion of other substances such as magnesium, sodium, and calcium. Fuller’s earth is a naturally occurring low-cost material, which is mainly used as an absorbing material for oil and grease, organic and mineral substances. However, Fuller’s earth in its natural form, even as granules, is very soft and can be crushed easily, which makes it unsuitable for use in an adsorption column.

Several studies [20,23] reported that magnetic chitosan is capable of removing strontium ions from aqueous solution, but interestingly pure chitosan was found to have negligible adsorption capacity for strontium [27]. Therefore, it was envisaged that Fuller’s earth can be dispersed onto the chitosan matrix wherein chitosan can be used as a binder. In this work, chitosan was coated on Fuller’s earth to form spherical beads. The bead is known as the CF bead (chitosan-Fuller’s earth bead) in this study. The CF bead was used to remove strontium from simulated low-level radioactive wastewater. The surface properties of the CF bead were characterized by surface charge, scanning electron micrograph (SEM), and X-ray photoelectron spectroscopy (XPS) analysis. The adsorption capacity of the CF beads for strontium was evaluated under both batch and dynamic conditions. The equilibrium adsorption data were correlated using the Langmuir isotherm equations. The sorption kinetics, isotherm data and dynamic breakthrough curves for strontium uptake were obtained to gain a better understanding of the adsorption process.

## 2. Materials and Methods

### 2.1. Materials

Fuller’s earth was obtained from Fisher Scientific Co., Hampton, NH, USA. The CF adsorbent was prepared as spherical beads using chitosan as a binder. The chitosan used in this study was 75 to 85% deacetylated and had a molecular weight of ~190,000–310,000 as determined from viscosity data by Aldrich Chemical Corporation. All chemicals used in this study were of analytical grade. A stock solution containing 1000 mg/L of strontium was prepared by dissolving strontium chloride in ultrapure water. The working solutions of various strontium concentrations were obtained by diluting the stock solution with ultrapure water.

### 2.2. Preparation of Chitosan-Fuller’s Earth (CF) Beads

Fuller’s earth powder of 35 mesh size was first soaked in 0.2 mol/L oxalic acid for 4 h. This helped to introduce various functional groups into Fuller’s earth, which in turn helped the adhesion of chitosan onto Fuller’s earth later during the bead making process. It was then washed with distilled water and dried in an oven at 60 °C for 12 h. Although washing with distilled water was not necessary, it was done to maintain a desired ratio of oxalic acid to chitosan which was found to be important for the performance of the beads as adsorbent. A higher concentration of oxalic acid would require a greater concentration of NaOH as well as a greater amount of water during the bead making process. An amount of 60 g of acid-washed Fuller’s earth was mixed with 30 g of chitosan flakes in a beaker with 1 L of 0.2 mol/L oxalic acid. The mixture was stirred for 4 h while heating at 313–323 K (40–50 °C) to obtain a homogeneous mixture. The spherical CF beads were prepared by drop-wise addition of the mixture into a 0.7 mol/L NaOH precipitation bath. The purpose of adding acidic Fuller’s earth-chitosan mixture to the NaOH solution was to assist rapid neutralization of oxalic acid so that the spherical shape of the bead could be retained. The CF beads were washed with deionized water to a neutral pH and oven dried for subsequent use.

### 2.3. Characterization

The surface area, pore volume, and pore diameter of the CF bead were evaluated by nitrogen adsorption/desorption at 77 K using the Brunauer–Emmett–Teller (BET) method (Micromeritics ASAP 2010 surface analyzer). The adsorbents were outgassed at 378 K under a vacuum of <10 μm Hg for a minimum period of 24 h. The equilibrium nitrogen adsorption data and the liquid nitrogen temperature in the relative pressure range of 0.05 to 0.30 atm (0.74 to 4.41 psia) were used to calculate the surface area. Scanning electron microscopy (SEM) (AMRAY 1600T) was used to investigate the surface morphology of the CF beads. The SEM micrographs were taken using backscatter electrons with an accelerating potential of 20 keV. The surface morphology of the CF bead was also investigated using a Philips EM 430 for scanning transmission electron microscopy (STEM). The microtome technique was used to prepare the sample for TEM analysis. X-ray photoelectron spectroscopy (XPS) is extremely useful for studying the valence shell and for chemical analysis of the variety of compounds. The XPS analysis of the CF bead before and after exposure to strontium ion was performed using a KRATOS model AXIS 165XPS spectrometer, with non-monochromatic magnesium X-rays (hν = 1253.6 eV) used as the excitation source at a power of 240 W. The spectrometer is equipped with an 8-channel hemispherical detector, and the pass energy of 5 to 160 eV was used during the analysis of samples. Each sample was exposed to x-rays for the same period of time and intensity.

### 2.4. Experimental Procedure

#### 2.4.1. Effect of pH on Strontium Adsorption onto CF Beads

The pH of the solution affects the degree of ionization, the surface charge, and the speciation of strontium, all of which can impact the adsorption mechanism and the uptake capacity of the adsorbent. The effect of pH on strontium removal from aqueous solutions was determined over the pH range of 2 to 11. The pH studies were carried out by exposing approximately 0.25 g of the CF beads to 100 mL of 20 mg strontium/L solution at 298 K. The pH of the solutions was adjusted by adding either 0.1 mol/L hydrochloric acid or 0.1 mol/L sodium hydroxide. The pH of the solutions was measured using a pH meter equipped with a glass electrode (Accumet, Fisher Scientific Co.).

#### 2.4.2. Batch Sorption Experiments of CF Beads

Equilibrium batch adsorption studies were carried out by exposing the beads to different concentrations of aqueous solutions of strontium in 125 mL Erlenmeyer flasks to a pre-determined pH and temperature. Approximately 0.25 g of the beads was added to 100 mL of solution. Such a quantity of the beads and solution ensured that an equilibrium condition was reached, i.e., all of the strontium was not adsorbed by the beads, which would have made it difficult to determine the equilibrium point. The flasks were placed in a constant temperature shaker bath for a specific time period. Following the exposure of the CF beads to strontium, the samples were collected at predetermined time intervals. The solutions were filtered and the filtrates were analyzed for strontium by an atomic absorption spectrometer (Perkin Elmer, Model 2380, Waltham, MA, USA). The adsorption isotherm at a particular temperature was obtained by varying the initial concentration of strontium ions. The amount of strontium adsorbed per unit mass of adsorbent ($Q_{e}$) was calculated using the following equation:

(1) $$Q_{e}=\frac{\left(C_{i}-C_{e}\right)V}{M}$$

where $C_{i}$ and $C_{e}$ represent initial and equilibrium concentrations in mg/L, respectively. V is the volume of the solution in liter (L) and M is the mass of the adsorbent in grams.

## 3. Results and Discussion

### 3.1. Characterization of CF Beads

**Figure 1:**
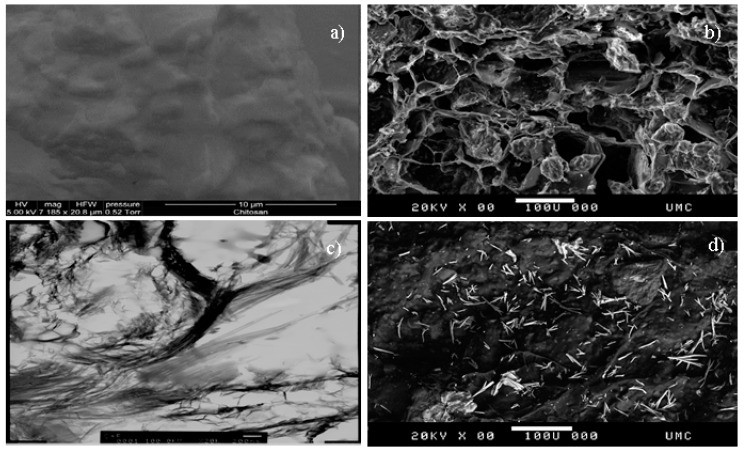
Scanning electron microscopy (SEM) micrograph: (**a**) pure chitosan bead, (**b**) CF bead, (**c**) Transmission electron microscopy (TEM) micrograph of the CF beads (the black spots represent Fuller’s earth particles), and (**d**) SEM micrograph of the CF beads after exposure to strontium solution.

The diameter of the vacuum oven-dried CF beads was about 2 mm. The SEM micrograph of the outer surface of the CF beads shown in Figure 1b appears to be porous in nature compared to pure chitosan beads (Figure 1a). A transmission electron (TEM) micrograph (Figure 1c) of a bead showed that a group of particles were lumped together and dispersed onto the chitosan film. Figure 1d shows the SEM micrograph of a CF bead following the adsorption of strontium. It appears that the surface morphology of the CF beads changed significantly following exposure to strontium solution. The CF beads became lumped after the adsorption of strontium. This may be due to a complex formation of strontium with the adsorbent. The strontium ion adsorbed onto the CF bead’s surface appeared as bright spike shapes as shown in Figure 1d.

**Figure 2:**
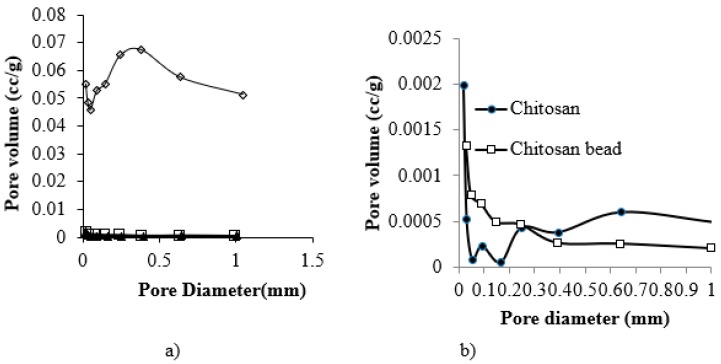
Pore size and pore volume distribution of (**a**) chitosan (•), the pure chitosan beads (□), and the CF beads (◊), and (**b**) chitosan (•) and the pure chitosan beads only.

The physical properties of the beads are given in Table 2. The BET (Brunauer–Emmett–Teller) surface areas of chitosan, Fuller’s earth, and the CF beads were found to be 1.36, 103.4, and 48.5 m^2^/g, respectively. Figure 2 shows the pore size and pore volume distributions of the CF beads, chitosan flake and the pure chitosan beads. The average diameter of the pores in the CF beads was found to be 0.02 μm, whereas the average pore diameter of chitosan flakes and the pure chitosan beads were 0.004 and 0.0047 μm, respectively. There are few pores in the CF beads, which were found to be in the 0.38 μm diameter range. The average pore volumes of chitosan flakes, pure chitosan beads and CF beads were found to be 1 × 10^−3^, 1.01 × 10^−3^, and 0.103 cm^3^/g respectively (Figure 2).

### 3.2. pH and Surface Charge Effects on Strontium Uptake by CF Beads

**Figure 3:**
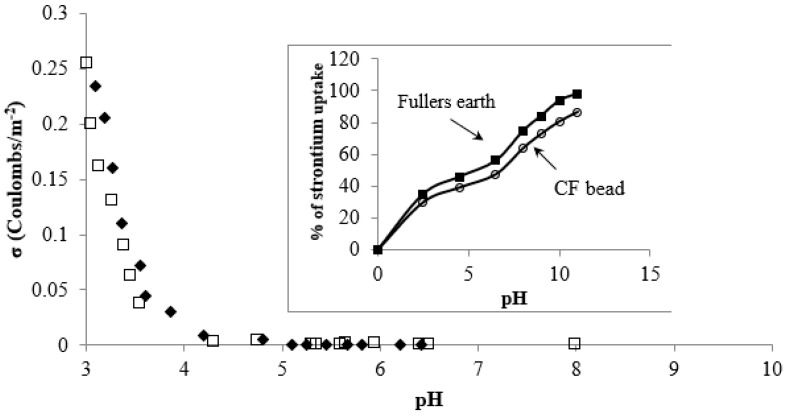
Surface charge analysis of the CF beads in (♦) 0.1 M and (□) 0.05 M NaNO_3_ solution. Effect of pH on strontium adsorption onto the CF beads (Inset).

The effect of pH on strontium uptake by the CF beads and Fuller’s earth is shown in Figure 3. It was observed that strontium uptake onto the CF beads increased with the increase in pH of the aqueous solution up to a value of 11.0. Both the CF beads and Fuller’s earth showed two distinct regions in the pH curve. The strontium uptake onto the CF bead is found to be lower in the solution pH ranges from 2 to 6 compared with the uptake of strontium in the pH range 7 to 11. This could be due to the competition of surface sites with H^+^ ion in the solution pH range, from 2 to 6.0. The increase of strontium uptake from pH 7 to 11 could be due to the formation of colloidal species which undergoes sedimentation. Therefore, experiments were not conducted at pH > 7. The increased capacity at pH > 7 may be a combination of both adsorption and precipitation on the surface. It is concluded that the beads had a maximum adsorption capacity at a pH of about 6.5, if the precipitated amount is not considered in the calculation. The surface charge of the CF bead was determined via a standard potentiometric titration method in the presence of a symmetric electrolyte (sodium nitrate) [28]. The surface charge of the CF bead was almost zero in the pH range of 6–8 (Figure 3). The point of zero charge (PZC) value of the CF beads was found to be 7.0. It may be noted that the pKa value of treated Fuller’s earth, which is known as hydrated alumina silicate, was determined to be ~7. Whereas, the pKa value of pure chitosan is found to be in the range 6.5–6.8. It was observed from Figure 3 that the protonation of the beads sharply increased at the pH range of 3–4.0, making the surface positive. At pH < 3.5, the difference between the initial pH and the pH after the equilibration time was not significant, suggesting complete protonation of the chitosan. At a higher pH (4.0–8.0), the surface charge of the bead slowly decreased, indicating slow protonation of chitosan on the bead. The PZC value of 7.0 and the behavior of the surface charge of the CF bead could have been due to the modification of chitosan when coated on Fuller’s earth, which makes it amphoteric in nature. It is mentioned elsewhere that the pure chitosan flake had an almost negligible adsorption capacity for strontium but pure Fuller’s earth produces pH curves similar to the CF bead (Figure 3). Therefore, it was assumed that the constituents of the Fuller’s earth were the active strontium binding sites for the CF beads. In aqueous solution, strontium can be present predominantly as non-hydrolyzed Sr^2+^ and SrOH^+^ species in a wide pH range [29], but SrOH^+^ is the main form of Sr^2+^ at pH > 12.8 [12,29]. From the surface charge analysis, the slow deprotonation of the CF bead surface is observed in the pH range of 4.5 to 7.0. At pH 6.5, the CF bead surface may form surface complexes with strontium ions (Sr^2+^) with the release of H^+^ ions. Since Fuller’s earth mainly consists of hydrated aluminum silicate, the formation of AlO^−^ and SiO^−^ on the CF bead surface is also therefore possible due to the deprotonation of the surface hydroxyl group. The H^+^ ion could be released from the CF bead surface into the solution as a result of ion exchange. Therefore, the Sr^2+^ adsorption may involve surface hydroxyls of the CF beads according to the ion-exchange mechanism [30,31]:

(2) $$-2S-\mathrm{OH}+{\mathrm{Sr}}^{2+}\Leftrightarrow \left({\mathrm{SO}}_{2}\right)\mathrm{Sr}+2H^{+}$$

(3) $$-S-\mathrm{OH}+{\mathrm{Sr}}^{2+}\Leftrightarrow -{\mathrm{SO}}^{-}{\mathrm{Sr}}^{2+}+H^{+}$$

### 3.3. Equilibrium Adsorption Studies

**Figure 4:**
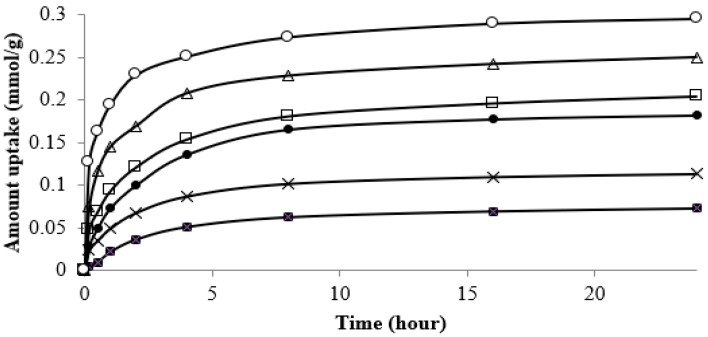
Effect of time and concentration on strontium adsorption onto the CF bead. The initial concentrations of the solution were (○) 1000, (∆) 500, (□) 280, (•) 220, (×) 100, and (■) 55 mg/L, respectively (pH: 6.5 and temperature: 298 K).

**Figure 5:**
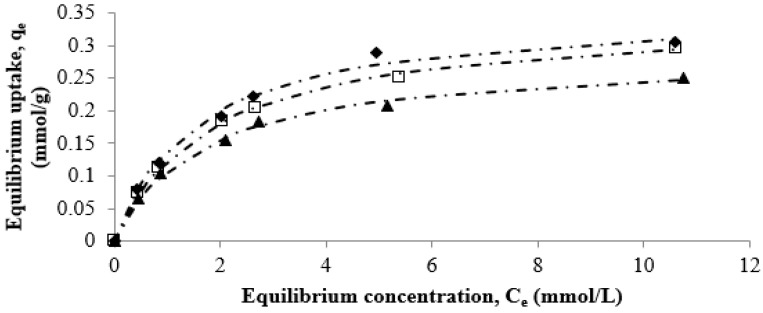
Effect of temperature on strontium adsorption onto the CF beads. The dashed lines are calculated from the one-site Langmuir equation, and the symbols (♦) 293 K, (□) 298 K, and (▲) 308 K represent experimental data.

As mentioned in the previous section, the maximum adsorption capacity of strontium on the CF beads occurred at a pH of 6.5 without any precipitation of strontium from the solution. Therefore, the equilibrium experiments were carried out at a pH of 6.5, if not stated otherwise. The sorption kinetics of strontium on the CF beads was determined in the concentration range of 0.63 mmol/L (55 mg/L) to 11.36 mmol/L (1000 mg/L). It was observed that the adsorption rate was time dependent. Almost 70% of strontium was adsorbed during the first 4 h of a run, and then the equilibrium was attained monotonically at 10 h in most of the runs. An exposure time of 24 h was used during batch studies to ensure that equilibrium was attained (Figure 4). The equilibrium studies for Sr^2+^ uptake by the CF beads at various temperatures were also performed at pH 6.5. The equilibrium adsorption data in the temperature range of 293–308 K appear to be of a Type I shape, suggesting a monolayer adsorption of strontium onto the CF bead (Figure 5).

The equilibrium data for Sr^2+^ uptake by the CF beads were fitted to the Langmuir isotherm equation. In general, the Langmuir isotherm provides a reasonable description of Type I systems and is often justified based on its ability to fit equilibrium data. The Langmuir equation can be expressed as:

(4) $$q_{e}=\frac{Q_{0}bC_{e}}{1+bC_{e}}$$

where $q_{e}$ = equilibrium uptake of adsorbate A by the adsorbent corresponding to concentration $C_{e}$; $Q_{0}$ = weight of adsorbate contained in the monolayer on the surface; $C_{e}$ = concentration of adsorbate in the fluid phase in equilibrium with the concentration $q_{e}$ on the solid; b = constant. The values of adsorption constants at various temperatures for the Langmuir isotherm are listed in Table 3. As can be seen from Table 3, the Langmuir equation provided good fit of the data with a maximum absolute error less than 1%.

### 3.4. Mechanism of Strontium Adsorption on CF Bead

**Figure 6:**
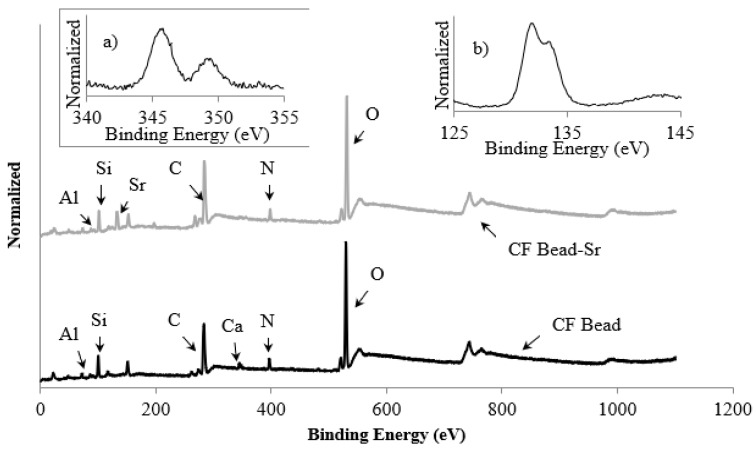
Survey scan of the CF bead exposed to strontium solution. Inset shows (**a**) calcium spectrum of the CF bead and (**b**) Sr-3d spectrum of the CF bead exposed to strontium solution.

The XPS analysis of the beads before and after the adsorption of strontium was used to gain a better understanding of the adsorption sites onto which strontium was adsorbed. To understand the binding of Sr^2+^ to the active sites on the CF bead, it was exposed to 100 mL of a 1.14 mmol/L strontium solution at pH 6.5. After 24 h of exposure, the beads were removed from the solution and dried at room temperature. Figure 6 (survey scan) shows the binding energies of various components in the CF bead and the CF bead exposed to strontium as noted during XPS analysis. In survey scans, the peaks were obtained at binding energies of 101, 73, 345.8 and 132 eV that correspond to Si-2p, Al-2p, Ca-2p and Sr-3d, respectively. The XPS spectrum (Figure 6) of the CF bead exposed to strontium showed the presence of Sr-3d peak at 131.9 eV with a FWHM of 3.43 eV, which on deconvolution showed two peaks (Figure 6b), one at 131.9 eV (Sr-3d5/2) and the other at 133.3 eV (Sr-3d1/2). Sr-3d photoelectrons contribute peaks at 131.9 eV and 133.3 eV that are the core level electrons and came essentially from the outer surface, while a small shoulder-like peak appears at 134.0 eV due to interaction with surface contaminants as suggested by Van der Heide 2002 [32].

**Figure 7:**
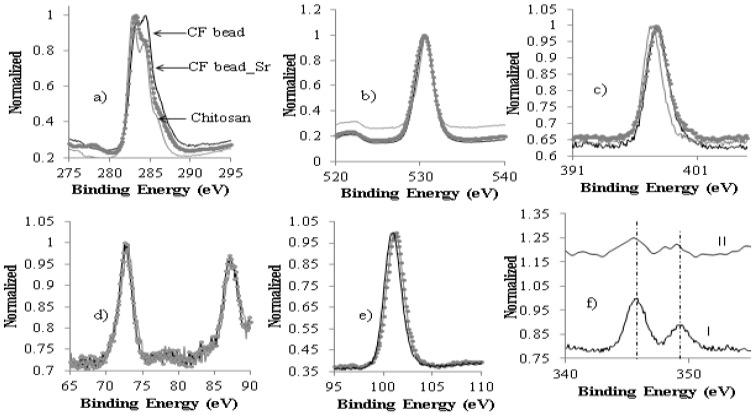
X-ray photoelectron spectroscopy (XPS) spectra of (**a**) C-1s, (**b**) O-1s, and (**c**) N-1s for the chitosan flake, CF bead, and the CF beads following exposure to strontium solution and of (**d**) Al-2p, and (**e**) Si-2p and (**f**) Ca-2p that present in the CF bead (i) before and (ii) after exposure to strontium solution.

The magnitude of the C-1s binding energy changed slightly only when the CF beads were exposed to strontium solution, whereas no significant change was observed for the O-1s and N-1s peaks (Figure 7a–c). The chemical shift is considered significant when they exceed 0.5 eV [33]. The magnitude of the binding energy shift depends on the concentration of different atoms, in particular on the surface of a material. Figure 7a shows that the C-1s peak reappears at 283 eV for the beads exposed to strontium solution, which is attributed to C–C or C–H linkage and indicates the strength of the surface interaction with strontium ion. Therefore, the spectra of C-1s and O-1s of the chitosan flake are similar to the spectra of the CF beads that were exposed to the strontium ion as they did not show any noticeable change in the position of binding energy. This indicates that the adsorption of strontium on the CF bead surface did not involve a noticeable electron transfer between the surface and adsorbate.

In comparison with the XPS (Figure 7), the binding energy of the N-1s, Si-2p, and Al-2p peaks of the CF beads did not shift before and after exposure to strontium solution but the relative intensities of these peaks were changed noticeably, indicating that the chemical state the of N, Si, and Al atoms was not changed after adsorption of strontium from the solution. Therefore, they did not take part in any adsorption processes. It has been reported that it is unlikely for Al and Si as skeletal elements to take part in any ion exchange processes [31]. The changes in relative intensity of the peaks may depend on the electronic configuration in the initial state. Figure 7f shows the calcium peak position in the survey scan of the CF bead before and after exposure to strontium solution. It is interesting to note that the Ca peak in the survey scan of the CF bead exposed to strontium is depleted with the appearance of the Sr peak at 131.9 eV compared to the survey scan of the CF bead (Figure 6). The binding energy of the Ca spectra at 345.8 eV and 349.4 eV for the CF bead that was exposed to strontium solution is shifted slightly and their relative intensity is also lower than the Ca-2p peaks of the CF bead (Figure 7f). This suggests that the partial replacement of Ca^2+^ from the CF bead may take place by Sr^2+^ during the adsorption process; therefore, Sr^2+^ uptake by the CF beads may occur following an ion-exchange process. Based on the sorption kinetic and XPS analysis, it was hypothesized that the adsorption mechanism of Sr^2+^ onto the CF beads surface follows an ion-exchange mechanism. The reaction is given as follows:

(5) $$-{\mathrm{SO}}^{-}{\mathrm{Ca}}^{2+}+{\mathrm{Sr}}^{2+}\Leftrightarrow -{\mathrm{SO}}^{-}{\mathrm{Sr}}^{2+}+{\mathrm{Ca}}^{2+}$$

### 3.5. Effect of Temperature on Strontium Uptake

The Gibbs free energy change equation is also used to gain insight into the thermodynamic nature of the strontium sorption process onto the CF beads. The Gibbs free energy change, ∆G, can be used to identify whether the chemical reaction that occurs during adsorption process is a spontaneous reaction or not. Therefore, it is considered as an important criterion for spontaneity. The Gibbs free energy equation for an adsorption process can be expressed as:

(6) $$∆G=-RTlnk_{c}$$

where $k_{c}$ is the sorption equilibrium constant, R is the gas constant, and T is the temperature (K). The dimensionless sorption equilibrium constant ($k_{c}$) can be calculated from:

(7) $$k_{c}=\frac{F}{\left(1-F\right)}$$

where F is the fraction attainment of metal ion sorbate at equilibrium. It was observed that more than 70% of strontium was adsorbed onto the CF bead within 4 h of exposure (Figure 4). Therefore, the values of the equilibrium constant ($k_{c}$) for the sorption of strontium ions were calculated at different temperatures and at an equilibrium time of 4 h using Equation (7).

Table 4 shows that the overall ∆G values for strontium uptake by the CF bead. The decrease in ∆*G* with temperature indicates more efficient adsorption at a higher temperature; therefore, it requires energy to carry out the sorption reaction process. The increase in $k_{c}$ values with the increases in sorption temperature suggests a strengthening of the adsorbate–adsorbent interaction at a higher temperature (Table 4). The rise in solution temperature may help more strontium ions to overcome the energy barrier, thereby to get attached to the CF bead surface, although rate data of strontium uptake (Table 5) as well as experimental strontium uptake at equilibrium show a trend that the uptake capacity decreases with the increase in temperature. This observation indicates that the total energy of the strontium ion increases with the increase in temperature and that creates an escaping tendency for the strontium ion from the surface of the resin at the equilibrium, thus reducing the strontium uptake capacity of the resin [34]. Both enthalpy and entropy factors can be estimated from the Gibbs free energy of the process. The Gibbs free change can be represented as follows:

(8) ∆G=∆H−T∆S

The values of enthalpy change (∆H) as well as entropy change (∆S) calculated from the intercept and slope of the plot of (∆G) versus T are also given in Table 6. The positive values of enthalpy and entropy reflect the complexities of this ion-exchange process [35]. The positive enthalpy (∆H) reveals energy is absorbed as ion exchange proceeds, and the reaction is said to be endothermic in nature. Saha and Chowdhury [36] reported that the heat evolved during physical adsorption is of the same order of magnitude as the heat of condensation, i.e., 2.1–20.9 kJ/ mol, while the heat of chemisorption generally falls into a range of 80–200 kJ/ mol. The value of ∆H for the adsorption of Sr^2+^ on the CF bead was found to be 8.287 kJ/mol (Table 4). Therefore, the adsorption of strontium on the CF bead can be physical in nature. The positive values of the entropy change (∆S) reveal that the freedom of metal ions is not too restricted in the CF bead [37]. Bilbao et al. [35] suggest that the positive values of entropy can be found when the sorbate is highly mobile, or dissociation reactions occur during the ion-exchange process, whereas Venkatesan et al. [38] suggest that the positive magnitudes of enthalpy and entropy changes indicate the endothermic nature of ion-exchange and the net transfer of water molecules from the sorbent to the solution. This phenomenon also suggests that the randomness at the solid–solution interface may increase with some structural changes in the adsorbate and adsorbent and an affinity of the CF bead towards strontium ions.

In Table 5, the rate constants for both first-order and second-order models were found to increase with the increase of solution temperature. The Arrhenius equation was applied to determine the activation energy of strontium uptake on the CF beads using the *K* values obtained from both kinetic models. The slope of the Arrhenius plots that is used to correlate the rate constant at different temperatures is as follows:

(9) $$k=k_{0}exp\left(\frac{-E}{RT}\right)$$

where E is activation energy; k is the rate constant of sorption, g/mg·min; $k_{0}$ is the temperature independent factor, g/mig·min; R is the gas constant, 8.314 J/mol·K; *T* is solution temperature, *K*.

**Figure 8:**
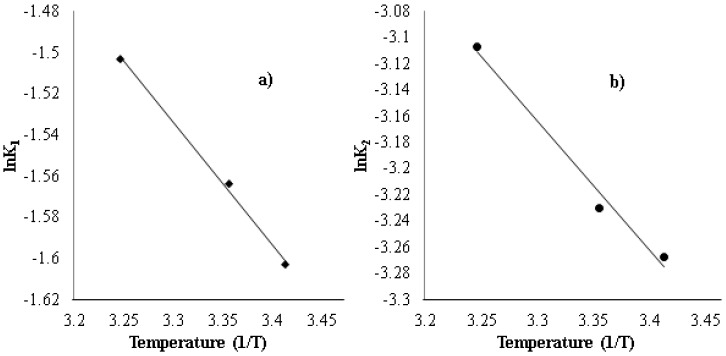
Arrhenius plots of (**a**) first-order (lnk_1_) and (**b**) second-order (lnk_2_) constants against reciprocal temperature for the adsorption of strontium onto the CF bead.

From the Arrhenius plots (Figure 8), it was observed that both first-order and second-order rate constants show a correlation coefficient value, $R^{2}$, of 99.7 and 98.6 respectively. The relationship between the k and T can be represented in an Arrhenius form as:

(9-1) $$k_{1}=0.434exp\left(\frac{-0.5963}{8.314T}\right)$$

(9-2) $$k_{2}=0.089exp\left(\frac{-0.986}{8.314T}\right)$$

From these equations, the rate constants of sorption are 0.434 g/mg.min and 0.089 g/mg·min for first-order and second-order kinetics. The activation energy plays a key role in the interaction between the solute and the sorbent. If the magnitude of the activation energy is between 4–42 kJ/mol, the adsorption mechanism is considered as chemisorption process [39]. The activation energy from first-order and second-order models is found to be 4.96 and 8.2 kJ/mol, respectively, which is in the range of activation energy of activated chemisorption. The ion-exchange process is a diffusion-controlled process, closer to physisorption than chemisorption. But at low activation energy, the adsorption rate is controlled by intraparticle diffusion mechanism and the process is governed by the interaction of physical nature [40]. The positive activation energy indicates minimum energy required to facilitate the forward ion-exchange process.

### 3.6. Kinetics Study of Strontium Adsorption

Adsorption kinetics data explain the rate of the adsorption of metal ions on the solid phase. For a batch contact process, the rate of sorption of strontium onto the CF bead surface is proportional to the amount of strontium uptake from the solution phase. A pseudo-first-order equation can be employed to describe reactions, whereas the pseudo-second-order equation affords the sorption capacity on the solid phase. Therefore, a pseudo-first-order or a pseudo-second-order model equation can be used to explain the kinetics of strontium sorption onto the CF beads. Linear forms of the first-order and second-order models, based on the adsorption equilibrium capacity, are as follows:

(10) log(qe−qt)=logqe−k1t2.303

(11) $$\frac{t}{q_{t}}=\frac{1}{k_{2}q_{e}^{2}}+\frac{1}{q_{e}}t$$

where $q_{e}$ is the amount of strontium uptake on CF bead at equilibrium and $q_{t}$ is the uptake at any time, t. The pseudo-first-order rate constant $k_{1}$ (min^−1^) is obtained by plotting $log\left(q_{e}-q_{t}\right)$ vs. t whereas the pseudo-second-order rate constant $k_{2}$ (g·mg^−1^·min) can be obtained by plotting $\frac{t}{q_{t}}$ versus t. The slope of the straight line of first-order and second-order kinetic plots provides the value of $k_{1}$ and $k_{2}$, respectively. The kinetic parameters obtained from Equations 10 and 11 are given in Table 6. It may be noted from Table 6 that at solution pH 6.5, the value of the first-order rate constant, $k_{1}$, remained almost constant, whereas the value of second-order rate constant, $k_{2}$, increased with the increase of solution concentration. It is also observed that the second-order model has higher correlation coefficient values for all the solution concentrations that were used in these studies compared with the data obtained by the first-order model. In addition, the second-order rate constant model provides a close match between the theoretical and experimental strontium uptake ($q_{e}$) by the CF beads. This suggests that strontium adsorption onto the CF beads follows the second-order model and that the overall rate of strontium adsorption on the CF bead may be controlled by a chemical process [41].

#### Elovich Equation

The Elovich equation was fitted to experimental data in order to investigate Sr^2+^ uptake on the CF bead. This equation was first proposed by Roginsky and Zeldovich; but it is commonly known as the Elovich equation and is extensively applied to chemisorption data [42]. The general expression of the Elovich equation is as follows:

(12) $$\frac{dq_{t}}{dt}=\alpha exp\left(-\beta q_{t}\right)$$

Integrating Equation 12 for the boundary conditions t=0 to t=t and $q_{t}=0$ to $q_{t}=q_{t}$, gives:

(13) $$q_{t}=\frac{1}{\beta }ln\left(\alpha \beta \right)+\frac{1}{\beta }ln\left(t\right)$$

where $q_{t}$ is the sorption capacity at any time, t (mg/g), α is the initial sorption rate (mg/g·min) and β is related to the extent of surface coverage and the activation energy for chemisorption (g/mg). The constants can be obtained from the slope and intercepts of the plot of $q_{t}$ vs. n(t). Table 6 shows the kinetic constants along with higher correlation coefficient (R^2^ > 96) value suggesting the applicability of Elovich equation.

Sorption kinetics of strontium on the CF beads using 220 mg/L (2.5 mM/L) strontium solution was evaluated at pH ~6.5 at different temperatures (293–308 K). The exposure time of 24 h was used during batch studies to ensure that equilibrium was attained. The data were fitted to the pseudo-first-order, pseudo-second-order, and Elovich equation. It was observed that the kinetic constants, $k_{1}$ and $k_{2},$ for both the first- and second-order processes increased with the increase in solution temperature (Table 5). With a higher correlation coefficient, the experimental data showed better agreement for the pseudo-second–order, rather than the first–order, model for the entire adsorption time at different temperatures. These observations also suggest that the strontium removal kinetics followed the pseudo-second-order model involving specific interaction between strontium and the CF bead surface [43,44].

In addition, the Weber–Morris model was also used to investigate the rate constant of the intra-particle transport of Sr^2+^ into the CF bead [45].

(14) $$q_{t}=k_{id}t^{1/2}+\theta$$

**Figure 9:**
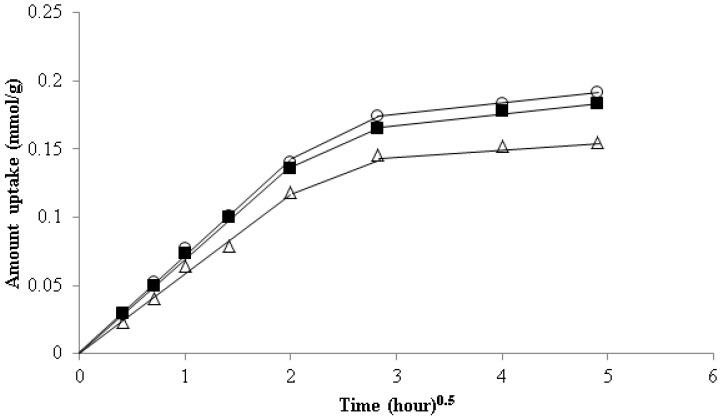
Intra-particle rate constant for strontium uptake onto the CF beads at temperatures: (○) 293 K, (■) 298 K, and (∆) 308 K.

In Equation (14), $q_{t}$ is the amount of strontium uptake (mg/g) at any time, t, $k_{id}$ is the rate constants of intra-particle transport (mg/g·min^1/2^) and θ (mg/g) is a constant related to the thickness of the boundary layer diffusion. If the intra-particle diffusion is the rate controlling step in the adsorption process, then the linear plot of $t^{1/2}$ vs. amount of strontium uptake, according to Weber–Morris model, should pass through the origin. Figure 9 shows a multilinear plot for the entire time range; therefore, it was assumed that there are various issues that influence the adsorption process. Based on the Weber–Morris plot, it is assumed that, in the first stage, the immobilization of strontium onto the CF bead surface occurs over time. As the surface sites become exhausted, the rate of intraparticle diffusion may control the adsorption process in the second stage. It is suggested that the rate of adsorption becomes relatively slow in the final removal stage due to the intra-particle diffusion of metal ions from the exterior to the interior sites of the adsorbent [43]. A similar observation was made by Min et al., 2016, for the removal of trace arsenate from water using functionalized chitosan nanofiber [46]. They reported that the first steep slope corresponds to the rate controlling stage as pore diffusion takes hold and the second moderate sorption stage is intra-particle diffusion control, influenced by the pore structure; finally, the slow sorption stage is related to strereo-hindrance effect derived from the adsorbed species. It is important to note that the plots do not pass through the origin as a slight deviation from the origin was observed at all temperatures (Figure 9). The intra-particle rate constant, $k_{id}$, was obtained from the slope of the straight-line portion of the plot of $q_{t}$ versus $t^{1/2}$ for various solution temperatures. It was observed that the straight line did not pass through the origin and the $k_{id}$ value decreased with the increase of solution temperatures (Table 5). This indicates that pore diffusion is not the sole rate controlling step; other mechanisms may control the rate of adsorption [47,48]. It is observed that the correlation coefficient, $R_{id}^{2}$ and $R_{Elovich}^{2}$, for the Weber–Morris and Elovich equation models is 98, whereas the correlation coefficient value for the pseudo-second-order equation is $R_{2}^{2}$ > 99 (Table 5). Therefore, it is hypothesized that either chemisorption or the pore diffusion process may dominate at any stage of the adsorption process [49].

The strontium sorption kinetics was further analyzed for the CF bead with radius, r, at any time, t, that can be expressed by the following the equation [50]:

(15) $$F=1-\frac{6}{\pi^{2}}\overset{\infty }{\underset{m=1}{\sum }}\frac{1}{m^{2}}exp\left(-\frac{Dm^{2}\pi^{2}t}{r^{2}}\right)$$

where $F=\frac{q_{t}}{q_{e}}$ is the fractional attainment of the strontium ion. The ability of Equation (15) to describe the sorption kinetics is limited due to the complex pore size, pore size distribution, particle size, and physicochemical changes such as swelling that may occur in the CF beads [51]. To investigate the function of diffusion in controlling the rate factor for the sorption of strontium onto the CF bead from aqueous solution, Equation (15) can be approximated to:

(16) (1−F)=6π2exp(−Dπ2tr2)

where (1−F) is the fraction of strontium uptake from the solution by the CF bead at any time (t). Therefore, in terms of the fraction of strontium remaining in the CF bead, Equation 16 can be rewritten as follows:

(17) $$ln\left(1-F\right)=ln\left(\frac{6}{\pi^{2}}\right)-\frac{D\pi^{2}t}{r^{2}}$$

Taking $ln\left(\frac{6}{\pi^{2}}\right)$ as a constant $\varphi_{r},$ so that it includes any anomalies arising out of factors such as swelling, and replacing $\frac{D\pi^{2}}{r^{2}}$ by a constant K, we can rewrite Equation (17) for a particle of radius r as:

(18) $$ln\left(1-F\right)=-Kt^{n}+\varphi_{r}$$

where n = 0.5 represents Fickian diffusion and 1 > n > 0.5 represents anomalous diffusion.

**Figure 10:**
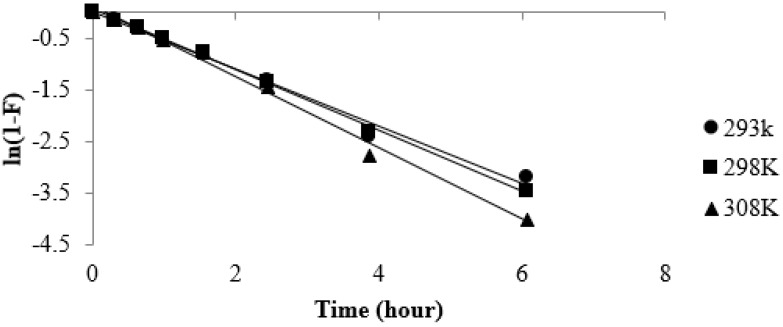
Effect of temperature on strontium diffusion onto the CF beads.

The diffusion co-efficient was estimated from the slope (Figure 10) and mean bead radius of 0.11 cm. For each temperature, the data were treated for n values 0.5 and 0.65 and it was observed that strontium uptake from the solution onto the CF bead showed a straight line when the n value was 0.65 (Figure 10). The function yield values range from 1.56 × 10^−7^ to 1.94 × 10^−7^ cm^2^/s for the particle diffusion coefficient for the temperature range of 293 K to 308 K.

## 4. Conclusions

Fuller’s Earth was coated successfully by chitosan and prepared as spherical beads with diameters of approximately 2 mm. The SEM micrograph reveals the heterogeneously porous nature of the beads. The equilibrium adsorption capacity of strontium was found to be dependent on solution pH. A one-site Langmuir model provided the best fit to the adsorption data for strontium. The maximum adsorption capacity of strontium onto the CF beads at a pH of 6.5 was found to be ~30 mg/g at 298 K. XPS analysis indicated that the constituent of Fuller’s earth, for example calcium, is the active metal exchange site for the CF beads. The ion-exchange reaction in fact differs from adsorption. Since nearly every ion-exchange process is accompanied by adsorption and desorption, adsorption is sometimes indistinguishable from ion exchange. It is evident from the surface charge and XPS analysis that strontium is removed from the aqueous solution by the CF bead through an ion-exchange reaction. The adsorption reaction may also occur at the hydrated oxide of the surface as the adsorption of strontium onto the CF bead was found to be pH dependent. The kinetic data suggest that the ion-exchange process follows anomalous diffusion. The negative ∆G value shows that the ion exchange of Sr^2+^ on the CF bead was feasible and spontaneous. The activation energy data according to the Arrhenius Equation suggest that the adsorption process was endothermic, and the process was governed by the interaction of physical nature. It is important to note that the regeneration of the column with the acidic solution causes swelling of the bead which may create hydrodynamic pressure build-up in the column. Future studies need to consider a suitable desorption solution in order to minimize swelling of the bead in the column.

## Figures and Tables

**Table 1:** Adsorption capacity of various adsorbents for strontium.

Adsorbent	Initial Concentration (mg/L)	pH	Exposure Time (h)	Temp. (K)	Uptake (mg/g)	Ref.
Natural attapulgite	5–300	4.8	5	303	8.11	[16]
Ca-alginate	10–500	Neutral	−	RT	6.7	[17]
PAN-Zeolite	25–175	5	−	298	0.011	[4]
Modified gibbsite	5–50	11.1	24	298	64.72	[8]
Almond shell-AC	45–102	Neutral	−	298	116.3	[18]
Composite sludge	−	10.25	2	318	23.04	[7]
Modified bentonite	25–100	4–8.5	3	298	46.1	[21]
Zr-MnO_2_/PAN	20–200	8	4	333	21.37	[19]
Natural clinoptilolite	−	7	−	−	9.8	[22]
Magnetic chitosan microsphere	5–300	8	5	303	81.96	[23]
Clinoptilolite/CoFe_2_O_4_	20–400	4	24	298	20.58	[24]
SBA-15	0–80	6	5	−	17.67	[25]
Chitosan-Fuller’s earth bead	20–1000	6.5	24	298	30.58	This study

**Table 2:** Physical properties of the Fuller’s earth beads.

Properties	Chitosan Fuller’s Earth Adsorbent
Particle diameter d_p_ (m)	2.2 × 10^−3^
Particle density ρ (kg/m^3^)	1395
Particle porosity (ε_p_)	2.2 × 10^−3^
Shape	spherical
Chitosan content (wt%)	32
Surface area (m^2^/g)	48.5
Pore volume (m^3^/kg)	1.03 × 10^−4^
Average pore diameter (m)	8.26 × 10^−9^

**Table 3:** Estimated parameters from Isotherm models.

Model	Parameters	Temperature		
		293 K	298 K	308 K
Langmuir isotherm	$q_{max}$ (mg/g)	31.15	30.03	25.13
	b (L/mg)	0.0074	0.0065	0.0069
	Absolute error (%)	−0.37	0.52	0.601
	Maximum (+) error	6.39	5.25	4.28
	Maximum (−) error	5.18	3.06	4.56

**Table 4:** Equilibrium constant and thermodynamic parameters at different temperatures.

Temperature (K)	K_c_	−∆G (kJ/mol)	∆H (kJ/mol)	∆S (kJ/mol·K)
293	2.72	2.44	8.2831	0.0365
298	2.833	2.58		
308	3.2	2.98		

**Table 5:** Rate parameters at different temperatures (condition: Initial concentration of Sr^2+^ in solution is 220 mg/L at pH 6.5).

Model	Parameters	Temperature (K)		
		293	298	308
1st-order	$k_{1}$ (min^−1^)	0.196	0.21	0.253
	$R_{1}^{2}$	95	97.4	99.5
2nd-order	$k_{2}\times 10^{-2}$ (g/mg·min)	3.71	3.9	4.64
	$R_{2}^{2}$	99.9	99.9	99.9
Elovich	α (mg/g·min)	32.43	31.864	26.123
	β (g/mg)	0.3153	0.33	0.38
	$R_{Elovich}^{2}$	98	98.12	96.92
Weber–Morris	$k_{1d}$ (mg/g·min^1/2^)	5.313	5.032	4.51
	θ (mg/g)	0.9753	1.085	0.613
	$R_{id}^{2}$	98.43	98.03	98.29

**Table 6:** The values for rate constants for the first-order and second-order rate constants.

Model	Parameters	Initial Concentration of Sr^2+^ in Solution (mg/L) at 298 K and pH 6.5					
		55	100	220	280	500	1000
1st-order	$q_{expt}$ (mg/g)	6.4	6.46	16.1	18	22	26
	$q_{theory}$ (mg/g)	5.14	5.91	11.57	11.48	11.22	10.96
	$k_{1}$ (min^−1^)	0.189	0.22	0.21	0.184	0.191	0.203
	$R_{1}^{2}$	97.04	98	97.4	97	94	97
2nd-order	$q_{theory}$ (mg/g)	7.14	7.09	17.1	18.73	22.52	26.5
	$k_{2}\times 10^{-2}$ (g/mg·min)	5.2	5.6	3.9	4.4	5.8	7.03
	$h_{0}$ (mg/g·min)	0.044	0.05	0.19	0.26	0.493	0.82
	$R_{2}^{2}$	99.8	99.3	99.9	99.9	99.94	99.96
Elovich	α (mg/g·min)	4.95	15.72	31.864	57.01	165.56	755.40
	β (g/mg)	0.698	0.519	0.33	0.3312	0.31	0.321
	$R_{Elovich}^{2}$	94	97.34	98.12	98.68	98.66	98.34
